# Shikonin Alleviates Gentamicin-Induced Renal Injury in Rats by Targeting Renal Endocytosis, SIRT1/Nrf2/HO-1, TLR-4/NF-κB/MAPK, and PI3K/Akt Cascades

**DOI:** 10.3390/antibiotics12050826

**Published:** 2023-04-28

**Authors:** Mohamed F. Balaha, Ahmed A. Alamer, Alaa A. Eisa, Hashim M. Aljohani

**Affiliations:** 1 Clinical Pharmacy Department, College of Pharmacy, Prince Sattam Bin Abdulaziz University, Al-Kharj 11942, Saudi Arabia; 2 Pharmacology Department, Faculty of Medicine, Tanta University, El-Gish Street, Tanta 31527, Egypt; 3 Department of Medical Laboratories Technology, College of Applied Medical Sciences, Taibah University, Medina 41477, Saudi Arabia; 4 Animal House Unit, King Fahd Medical Research Center, King Abdulaziz University, Jeddah 21589, Saudi Arabia; 5 Department of Clinical Laboratory Sciences, College of Applied Medical Sciences, Taibah University, Madina 41477, Saudi Arabia; 6 Department of Pathology and Laboratory Medicine, College of Medicine, University of Cincinnati, Cincinnati, OH 45221, USA

**Keywords:** shikonin, gentamicin-induced renal injury, renal endocytosis, SIRT1/Nrf2/HO-1, TLR-4/NF-κB/MAPK, PI3K/Akt

## Abstract

Gentamicin causes kidney injury due to its accumulation in proximal tubule epithelial cells via the megalin/cubilin/CLC-5 complex. Recently, shikonin has been shown to have potential anti-inflammatory, antioxidant, antimicrobial, and chloride channel-inhibiting effects. The current study investigated the alleviation of gentamicin-induced renal injury by shikonin while preserving its bactericidal effect. Nine-week-old Wistar rats were administered 6.25, 12.5, and 25 mg/kg/day shikonin orally, one hour after the i.p. injection of 100 mg/kg/day gentamicin for seven days. Shikonin significantly and dose-dependently alleviated gentamicin-induced renal injury, as revealed by restoring normal kidney function and histological architecture. Furthermore, shikonin restored renal endocytic function, as indicated by suppressing the elevated renal megalin, cubilin, and CLC-5 and enhancing the reduced NHE3 levels and mRNA expressions induced by gentamicin. These potentials could be attributed to the modulation of the renal SIRT1/Nrf2/HO-1, TLR-4/NF-κB/MAPK, and PI3K/Akt cascades, which enhanced the renal antioxidant system and suppressed renal inflammation and apoptosis, as indicated by enhancements of SIRT1, Nrf2, HO-1, GSH, SOD, TAC, Iκb-α, Bcl-2, PI3K, and Akt levels and mRNA expressions, with reduction of TLR-4, NF-κB, MAPK, IL-1β, TNF-α, MDA, iNOS, NO, cytochrome c, caspase-3, Bax levels, and Bax/Bcl-2 ratio. Therefore, shikonin is a promising therapeutic agent for alleviating gentamicin-induced renal injury.

## 1. Introduction

Despite new antibiotic generations, aminoglycosides are still widely used to treat a broad spectrum of Gram-negative bacterial infections. Gentamicin is a well-known aminoglycoside antibiotic due to its broad spectrum, prompt bactericidal activity, low resistance rate, and inexpensive cost [1]. Nonetheless, its use has been limited by its renal injurious side effects in 10–20% of treatment regimens, reaching 50% after 14 days of therapy [2]. Renal injury is, therefore, a clinical concern that causes higher comorbidity during and after gentamicin therapy and can progress to acute kidney failure (AKI); hence, kidney function should be checked regularly [3]. Despite introducing drugs with fewer side effects, the synergistic action of aminoglycosides with other antibiotics and the emergence of multidrug resistance has led to the reconsideration of treatment with aminoglycosides as a reasonable alternative [4].

Although the mechanisms underlying gentamicin-induced renal injury remain unclear, gentamicin accumulation in proximal tubule epithelial cells is a significant component of gentamicin-induced kidney injury [1]. Gentamicin entry and accumulation rely on a distinct protein and cation transport mechanism in the proximal tubules due to its hydrophilic characteristics, which inhibit penetration through the renal cell membrane [5]. The presence of megalin, cubilin, 2Cl/H^+^-exchanger (ClC-5), and Na^+^/H^+^ exchanger isoform 3 (NHE3) is required for endocytosis in the proximal tubules [2,6,7]. Furthermore, megalin knockout significantly reduced gentamicin uptake, indicating that endocytosis is the principal pathway for gentamicin accumulation in the kidney and, hence, its nephrotoxic impact [8]. Gentamicin is taken by endocytosis and transported to the Golgi bodies, endoplasmic reticulum, and lysosomes after binding to the megalin/cubilin complex, destabilizing lipid metabolism, resulting in gentamicin-induced kidney injury and cell death [9]. Furthermore, gentamicin released into the cytoplasm upon membrane rupture activates proapoptotic proteins that directly damage the mitochondria, prompting apoptosis, the generation of reactive oxygen species (ROS), reactive nitrogen species (RNS), and an inflammatory response, which contribute to the development and exacerbation of renal injury [1,2,10,11]. As a result, modulating the endocytosis pathway without altering gentamicin’s antibacterial activity is a reasonable approach for overcoming gentamicin-induced kidney injury while retaining its bactericidal capacity.

Shikonin, (5,8-dihydroxy-2-[(1R)-1 hydroxy-4-methylpent-3-enyl] naphthalene-1,4 dione), isolated mainly from Lithospermum Erythrorhizon. Shikonin has recently attracted attention due to its potential multiple pharmacological activities, including anti-inflammatory, anti-cancer, cardioprotective, antimicrobial, analgesic, and neuroprotective potentialities [12]. In addition, shikonin also demonstrated nephroprotective properties in diabetic nephropathy and lethal endotoxemia animal models by activating the renal nuclear factor-erythroid factor 2-related factor 2 (Nrf2) cascade and the antioxidant defense system, as well as decreasing circulating pro-inflammatory cytokines (Interleukin (IL)-6 and tumor necrosis factor (TNF)-α), and HIF-1 expression, along with inhibiting apoptosis, PKM2, NLRP3/caspase-1/IL-1β inflammasome, and improving animal survival [13,14,15,16,17]. Moreover, Shikonin is a chloride channels (CLC) inhibitor [18], so we hypothesized that it might suppress renal ClC-5, essential in megalin endocytosis, thus reducing endocytic function induced by gentamicin.

As a result, we investigated the role of shikonin in alleviating gentamicin-induced renal injury in rats while maintaining its bactericidal effect, emphasizing its potential modulating activities on renal endocytosis, SIRT1/Nrf2/HO-1, TLR-4/NF-κB/MAPK, and PI3K/Akt cascades, as well as renal oxidative stress, apoptosis, inflammation, and histopathological changes induced by repeated gentamicin injection.

## 2. Results

### 2.1. Shikonin Mitigates Gentamicin-Triggered Renal Dysfunction

All renal function indices assessed in serum and urine, and the KSI were not statistically different between the control and SKN groups. However, serum Cr, BUN, cystatin-C, renal tissue KIM-1, urine volume, flow rate, and cystatin-C levels, as well as the KSI, were significantly increased, while urine creatinine level, as well as the GFR, were significantly reduced in the GTM group compared to the CON group. Furthermore, compared to the GTM group, the changes in all renal function parameters were recovered in the shikonin-treated rats in a dose-dependent manner, with the SKH group showing a more significant restoration of the renal function parameters than the SKL and SKM groups (Table 1).

### 2.2. Shikonin Restored the Endocytosis Function of Proximal Tubule Epithelial Cells Stimulated by Gentamicin

**Figure 1:**
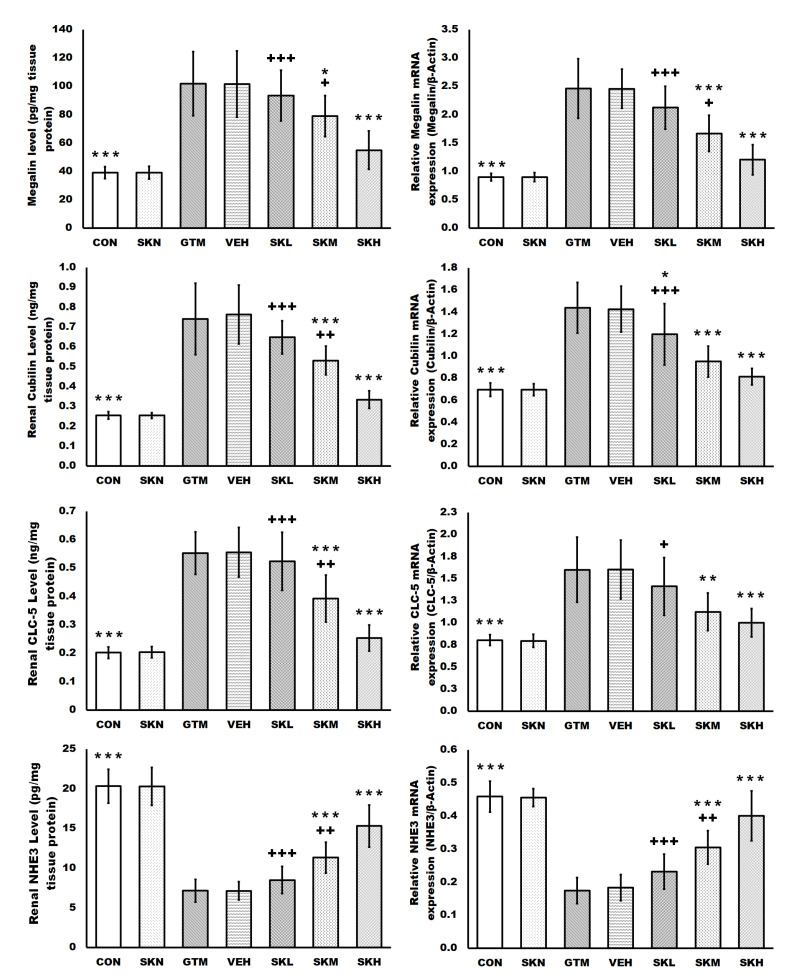
Shikonin restored the endocytosis function of proximal tubule epithelial cells stimulated by gentamicin. The results were expressed as mean ± SD. CON, normal rats administered PBS i.p., SKN, the negative control group, which were orally treated with 25 mg/kg/day of shikonin dissolved in 1% DMSO for seven days, one h after the PBS injection, GTM, renal injury-induced rats by i.p. injection of 100 mg/kg/day gentamicin for seven days, VEH, renal injury-induced rats, which were orally treated with 1% DMSO in PBS (vehicle of shikonin), SKL, SKM, and SKH, renal injury-induced rats, which were orally treated with 6.25, 12.5, and 25 mg/kg/day of shikonin for seven days, respectively, one h after the gentamicin injection. * *p* < 0.05, ** *p* < 0.01, and *** *p* < 0.001 (vs. GTM group), + *p* < 0.05, ++ *p* < 0.01, and +++ *p* < 0.001 (vs. SKH group).

The current study assessed the endocytosis function of proximal tubule epithelial cells by measuring renal megalin, cubilin, ClC-5, and NHE3 protein levels and mRNA expression, as well as urine excretion of megalin ligands, albumin, and calcium. Repeated gentamicin injections significantly increased megalin, cubilin, and ClC-5 protein levels and mRNA expression, as well as urine albumin and calcium levels, while significantly decreasing NHE3 protein levels and mRNA expression compared to the control group. However, concurrent treatment with shikonin significantly decreased the protein levels and mRNA expressions of megalin, cubilin, and ClC-5, as well as urine albumin and calcium levels, while significantly increasing the NHE3 protein levels and mRNA expression in a dose-dependent manner compared to the GTM group. Additionally, only the shikonin treatment of normal rats resulted in non-significant differences in the endocytosis function of proximal tubule epithelial cells compared to the CON group (Figure 1 and Table 1).

### 2.3. Shikonin Defended against Renal Oxidative Stress and Activated the SIRT1/Nrf2/HO-1 Cascades in Rats with Gentamicin-Induced Renal Damage

**Figure 2:**
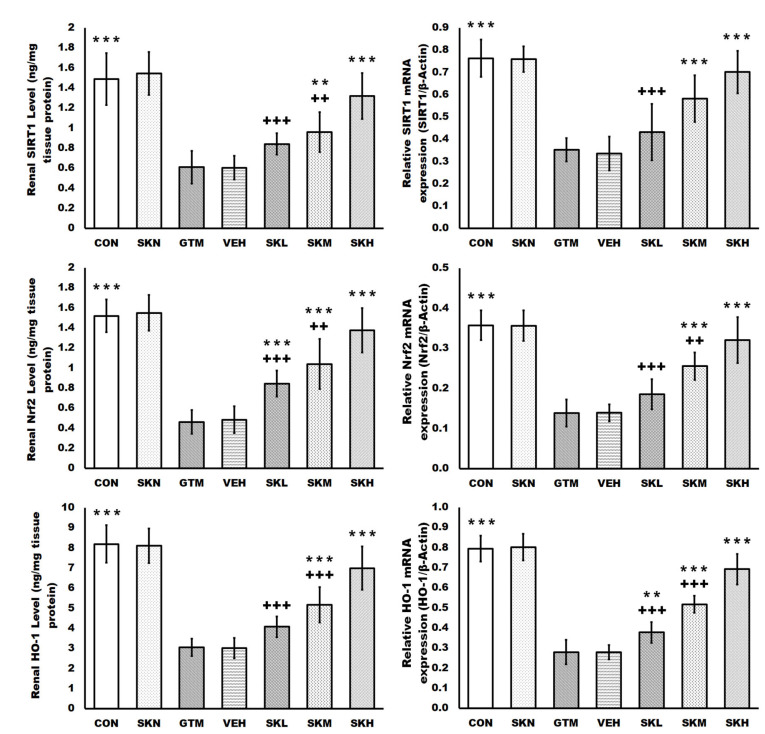
Shikonin activated the SIRT1/Nrf2/HO-1 cascades in rats with gentamicin-induced renal injury. The results were expressed as mean ± SD. CON, normal rats administered PBS i.p., SKN, the negative control group, which were orally treated with 25 mg/kg/day of shikonin dissolved in 1% DMSO for seven days, one h after the PBS injection, GTM, renal injury-induced rats by i.p. injection of 100 mg/kg/day gentamicin for seven days, VEH, renal injury-induced rats, which were orally treated with 1% DMSO in PBS (vehicle of shikonin), SKL, SKM, and SKH, renal injury-induced rats, which were orally treated with 6.25, 12.5, and 25 mg/kg/day of shikonin for seven days, respectively, one h after the gentamicin injection. ** *p* < 0.01 and *** *p* < 0.001 (vs. GTM group), ++ *p* < 0.01, and +++ *p* < 0.001 (vs. SKH group).

**Figure 3:**
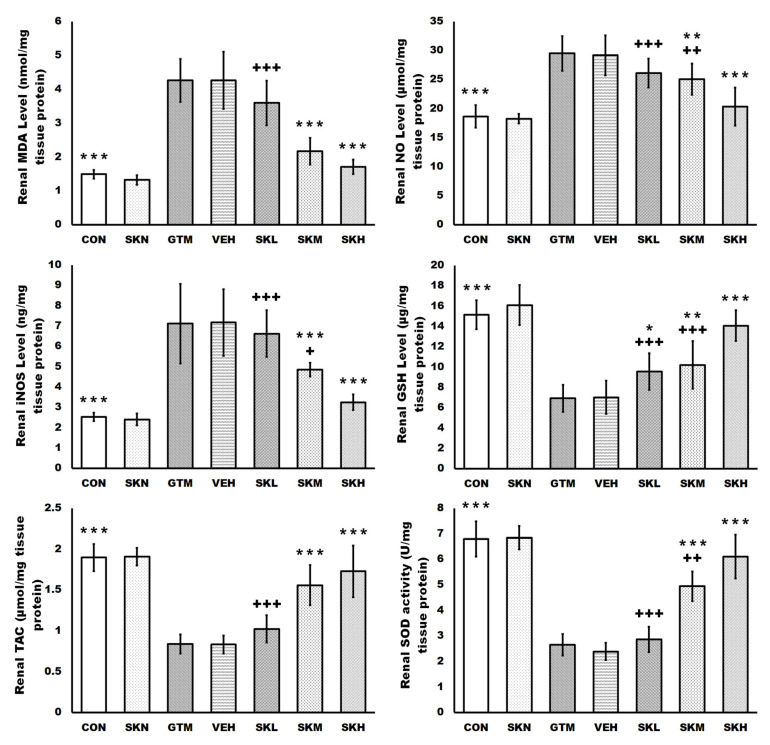
Shikonin defended against renal oxidative stress in rats with gentamicin-induced renal injury. The results were expressed as mean ± SD. CON, normal rats administered PBS i.p., SKN, the negative control group, which were orally treated with 25 mg/kg/day of shikonin dissolved in 1% DMSO for seven days, one h after the PBS injection, GTM, renal injury-induced rats by i.p. injection of 100 mg/kg/day gentamicin for seven days, VEH, renal injury-induced rats, which were orally treated with 1% DMSO in PBS (vehicle of shikonin), SKL, SKM, and SKH, renal injury-induced rats, which were orally treated with 6.25, 12.5, and 25 mg/kg/day of shikonin for seven days, respectively, one h after the gentamicin injection. * *p* < 0.05, ** *p* < 0.01, and *** *p* < 0.001 (vs. GTM group), + *p* < 0.05, ++ *p* < 0.01, and +++ *p* < 0.001 (vs. SKH group).

Gentamicin injections caused severe oxidative stress in the renal tissues as compared to the CON group, as evidenced by significantly higher renal levels of MDA, NO, and iNOS. Furthermore, gentamicin decreased renal antioxidant defenses, as seen by significantly reduced GSH, TAC levels, and SOD activity, as well as SIRT1, Nrf2, and HO-1 protein levels and mRNA expression. In contrast, shikonin treatment in gentamicin-induced renal injury rats restored these oxidative abnormalities, as evidenced by a dose-dependent significant decrease in the renal MDA, NO, and iNOS levels compared to the GTM group. Meanwhile, shikonin increased the renal antioxidant array dose-dependently, as evidenced by a significant rise in GSH, TAC levels, and SOD activity, as well as SIRT1, Nrf2, and HO-1 protein levels and mRNA expression compared to the GTM group. Furthermore, only shikonin administration to normal rats resulted in non-significant changes in these indicators compared to the CON group (Figure 2 and Figure 3).

### 2.4. Shikonin Amended the Gentamicin-Induced Renal Inflammatory Signals

**Figure 4:**
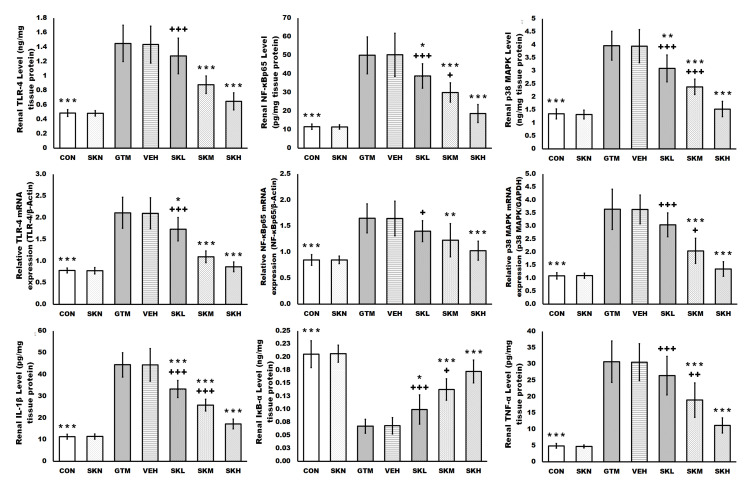
Shikonin amended the gentamicin-induced renal inflammatory signals. The results were expressed as mean ± SD. CON, normal rats administered PBS i.p., SKN, the negative control group, which were orally treated with 25 mg/kg/day of shikonin dissolved in 1% DMSO for seven days, one h after the PBS injection, GTM, renal injury-induced rats by i.p. injection of 100 mg/kg/day gentamicin for seven days, VEH, renal injury-induced rats, which were orally treated with 1% DMSO in PBS (vehicle of shikonin), SKL, SKM, and SKH, renal injury-induced rats, which were orally treated with 6.25, 12.5, and 25 mg/kg/day of shikonin for seven days, respectively, one h after the gentamicin injection. * *p* < 0.05, ** *p* < 0.01, and *** *p* < 0.001 (vs. GTM group), + *p* < 0.05, ++ *p* < 0.01, and +++ *p* < 0.001 (vs. SKH group).

Gentamicin injections resulted in a significant increase in a variety of inflammatory signal-mediated renal injury mechanisms, with gentamicin increasing renal TLR-4, NF-κBp65, p38 MAPK protein levels and mRNA expressions, and IL-1β, and TNF-α levels, while decreasing renal IκB-α level, when compared to the CON group. However, animals treated with shikonin significantly and dose-dependently suppressed these inflammatory signals in renal tissues when compared to the GTM group, as revealed by a reduction in increased renal TLR-4, NF-κBp65, and p38 MAPK protein levels and mRNA expressions, and IL-1β, and TNF-α levels, as well as an increase in reduced renal IκB-α level induced by gentamicin injections. Moreover, shikonin administration in the SKN group did not differ significantly from the CON group regarding renal tissue inflammatory signals (Figure 4).

### 2.5. Shikonin Inhibited the Gentamicin-Induced Renal Apoptosis

**Figure 5:**
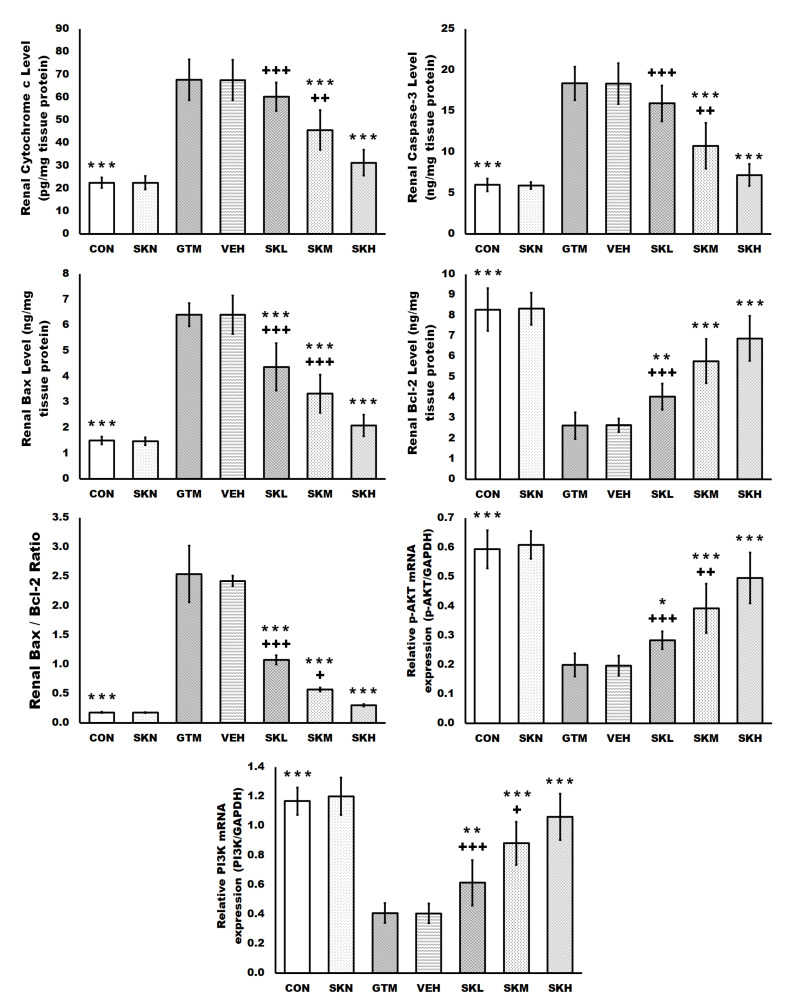
Shikonin inhibited gentamicin-induced renal apoptosis. The results were expressed as mean ± SD. CON, normal rats administered PBS i.p., SKN, the negative control group, which were orally treated with 25 mg/kg/day of shikonin dissolved in 1% DMSO for seven days, one h after the PBS injection, GTM, renal injury-induced rats by i.p. injection of 100 mg/kg/day gentamicin for seven days, VEH, renal injury-induced rats, which were orally treated with 1% DMSO in PBS (vehicle of shikonin), SKL, SKM, and SKH, renal injury-induced rats, which were orally treated with 6.25, 12.5, and 25 mg/kg/day of shikonin for seven days, respectively, one h after the gentamicin injection. * *p* < 0.05, ** *p* < 0.01, and *** *p* < 0.001 (vs. GTM group), + *p* < 0.05, ++ *p* < 0.01, and +++ *p* < 0.001 (vs. SKH group).

Renal cytochrome c, caspase-3, Bax, and Bcl-2 protein levels, the Bax/Bcl-2 ratio, and p-AKT and PI3K mRNA expression were not statistically different between the CON and SKN groups. However, renal cytochrome c, caspase-3, Bax protein levels, and the Bax/Bcl-2 ratio were significantly increased in the gentamicin group’s kidneys, but Bcl2 protein levels and mRNA expression of p-AKT and PI3K were significantly lowered. These changes were significantly and dose-dependently reversed in the kidneys of shikonin-treated groups compared to the GTM group, as evidenced by a significant decrease in the increased renal cytochrome c, caspase-3, and Bax protein levels, as well as the Bax/Bcl-2 ratio, with a significant increase in the reduced renal Bcl2 protein level and mRNA expression of p-AKT and PI3K, induced by repeated gentamicin injections (Figure 5).

### 2.6. Shikonin Augmented the Antibacterial Activity of Gentamicin

**Figure 6:**
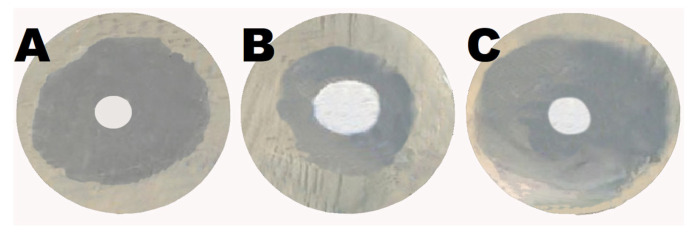
Shikonin augmented the antibacterial activity of gentamicin: (**A**) the well containing 10 mg gentamicin demonstrated a growth-inhibition zone against *E. coli* of 28 mm, (**B**) the well containing 10 mg shikonin showed a growth-inhibition zone against *E. coli* of 15 mm, (**C**) the well containing 5 mg gentamicin and 5 mg shikonin had a 33 mm inhibitory zone against *E. coli*.

Using the agar diffusion technique, Shikonin boosted the antibacterial activity of gentamicin. A 15 mm growth-inhibition zone against *E. coli* was observed in the well containing 10 mg shikonin. Meanwhile, the gentamicin-containing well demonstrated an inhibitory zone of 28 mm. On the other hand, the well containing 5 mg gentamicin and 5 mg shikonin had a 33 mm inhibitory zone. These data show that shikonin enhanced gentamicin’s antibacterial activity (Figure 6).

### 2.7. Shikonin Reversed the Renal Histopathological Alterations Induced by Gentamicin Injection

**Figure 7:**
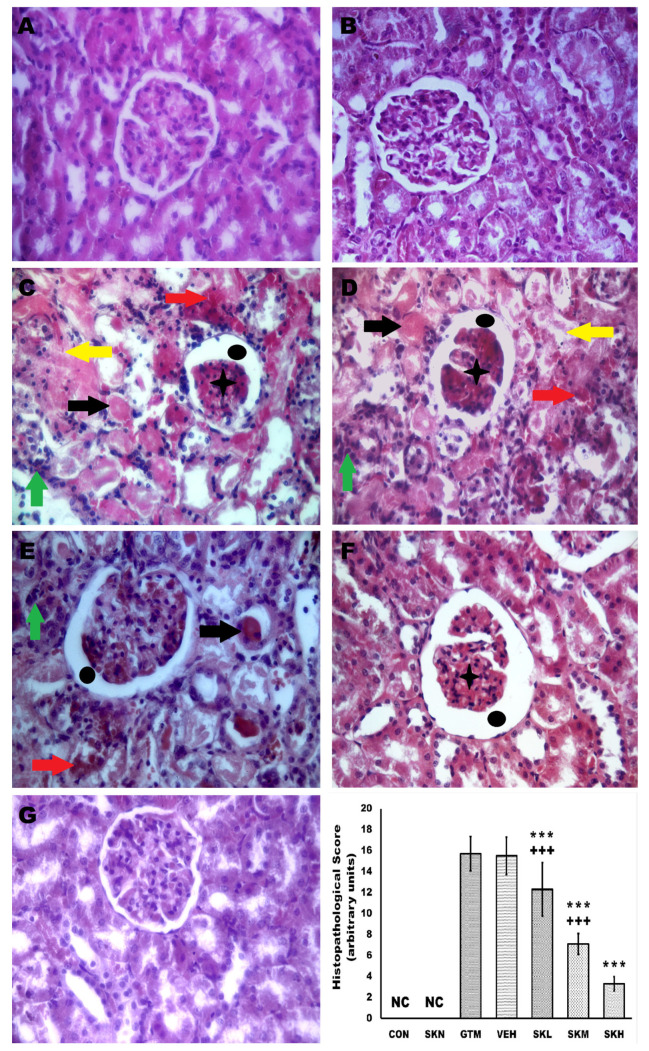
Shikonin demotes the renal tissue’s histopathologic changes and histopathological score in gentamicin-induced renal injury. (**A**,**B**) The CON and SKN groups’ renal tissue demonstrated an intact renal morphology with normal renal corpuscles and tubules. (**C**,**D**) GTM, and VEH groups showed that gentamicin administration caused tubular necrosis, cast formation, leukocytic infiltration, Bowman’s capsule space widening, congested glomerular and interstitial blood capillaries, and a significant rise in the histopathological score as compared to the CON group. (**E**–**G**) Renal injury-induced rats, which were orally treated with 6.25, 12.5, and 25 mg/kg/day, respectively, showed that shikonin administration dose-dependently and significantly conserved renal architecture and reduced these pathologic alterations compared to the GTM group, to be like the CON group in the SKH group. Yellow arrow, tubular necrosis, black arrow, cast formation, green arrow, leukocytic infiltration, black circle, Bowman’s capsule space widening, black asterisk, congested glomeruli, and red arrow, congested interstitial blood capillaries, *** *p* < 0.001 (vs. GTM group), and +++ *p* < 0.001 (vs. SKH group) (H&E, X400).

Histopathological examination was used to further analyze microscopic changes in rats’ kidneys to illustrate shikonin’s positive effect. The CON and SKN groups’ renal tissue demonstrated an intact renal morphology with normal renal corpuscles and tubules. However, gentamicin administration caused tubular necrosis, cast formation, leukocytic infiltration, Bowman’s capsule space widening, congested glomerular and interstitial blood capillaries, and a significant rise in the histopathological score as compared to the CON group. Remarkably, shikonin administration dose-dependently and significantly conserved renal architecture and reduced these pathologic alterations compared to the GTM group, to be like the CON group in the SKH group. These data show that shikonin alleviated gentamicin-induced kidney histopathologic alterations (Figure 7).

## 3. Discussion

Gentamicin is a potent antibiotic used to treat severe bacterial infections; nevertheless, it is commonly associated with side effects, most notably kidney damage. In the most seriously afflicted individuals, gentamicin-induced kidney injury is linked with proximal tubule epithelial cell dysfunction within the renal cortex, which may lead to renal failure. In addition, gentamicin accumulation in renal tissue causes inflammation, apoptosis, and oxidative damage, all of which contribute to the development of kidney injury. As a result, molecules that inhibit excessive oxidative stress, apoptosis, and inflammation may protect against gentamicin-induced kidney injury [1,2]. Data from the current study revealed that shikonin was able to alleviate gentamicin-induced renal injury in rats while maintaining its bactericidal effect by modulating renal endocytosis, SIRT1/Nrf2/HO-1, TLR-4/NF-κB/MAPK, PI3K/Akt cascades, thus abrogating renal oxidative stress, apoptosis, inflammation, and histopathological changes induced by repeated gentamicin injection.

Gentamicin uptake via endocytosis results in its accumulation in proximal tubule lysosomes, causing lysosomal membrane rupture and, as a result, tubular cell death [1]. The megalin/cubilin complex and their modulating molecules, CLC-5 and NHE3, are thought to be the basis for gentamicin uptake and accumulation in proximal tubular epithelial cells [19,20,21]. In the present study, megalin, cubilin, and CLC-5 renal levels and mRNA expressions were elevated in gentamicin-treated rats; however, the NHE3 level and mRNA expression were reduced in the gentamicin-treated group. The increased expression of the megalin/cubilin complex increased the proximal tubular uptake of gentamicin and its accumulation in the Golgi apparatus and lysosomes, which, when reached at a particular concentration, induces their rupture with the release of its content into the cytoplasm, causing oxidative stress and apoptosis and thus promoting the development and progression of renal injury [22]. On the other hand, treatment with shikonin significantly and dose-dependently decreased megalin/cubilin complex expression, which decreased gentamicin accumulation in proximal tubular cells and protected the kidneys from damage caused by gentamicin.

Additionally, ClC-5 is a Cl^−^/H^+^ transporter expressed chiefly in the kidney, where it aids in endosome acidification, which is essential for proximal tubular uptake and trafficking. ClC-5’s unique role in megalin and cubilin-mediated endocytosis was demonstrated when ClC-5 knockout animals showed lower endocytosis of megalin/cubilin ligands, suggesting that ClC-5 is required for megalin and cubilin trafficking in proximal tubules. Moreover, the acidification of endosomes enhances dissociation between megalin, and its ligand, followed by megalin recycling to the brush border membrane [1,7,22]. Our findings showed that shikonin inhibited megalin/cubilin complex expression in gentamicin-treated rats in a dose-dependent manner. This impact might be related to ClC-5 expression downregulation. Shikonin’s action on renal ClC-5 is consistent with its previously described activity as a CLC inhibitor [18].

Moreover, NHE3 is the most common isoform of Na^+^/H^+^ exchanger discovered in proximal tubular cells, where it promotes luminal isotonic reabsorption of Na^+^ across the membrane. NHE3 also reabsorbs amino acids and calcium from urine filtrate [7,23]. Moreover, after endocytosis with megalin, NHE3 is thought to use the outward trans-vesicular Na^+^ gradient of endocytic vesicles and early endosomes to promote the inward H^+^ flow necessary for endosomal acidification, which is a critical step in megalin trafficking [7]. In the current study, NHE3 level and mRNA expression were significantly reduced after gentamicin treatment. These findings are consistent with previously published studies showing that gentamicin therapy decreased GFR by decreasing sodium transporter function in the proximal nephron via NHE3 downregulation [23]. Furthermore, it is thought that the increased expression of CLC-5 compensates for the reduced acidification of the megalin/cubilin complex caused by NHE3 downregulation. However, shikonin treatment significantly and dose-dependently enhanced the reduced NHE3 level and mRNA expression induced by repeated gentamicin injections, restoring regular acidification activity of the proximal tubular cells while downregulating CLC-5. Further, it is well known that NHE3 activity is increased with PI3K cascade activation; in the current study, shikonin treatment of the gentamicin-injected groups increased PI3K expression, which may play a role in the augmentation of NHE3 level and mRNA expression [24].

Additionally, urine analysis revealed increased urinary excretion of albumin and calcium, which are megalin/cubilin ligands, in the gentamicin group despite the overexpression of the megalin/cubilin complex [1,23,25]. That could be attributed to the interference of gentamicin with cations and amino acids for the uptake by the megalin/cubilin complex in renal tubular cells, and thus urinary albumin and calcium excretion increase [1,5,7]. Likewise, the reduced expression of NHE3, which is partly responsible for the impaired reabsorption of albumin and calcium, is thought to be responsible for the increased urine albumin and calcium in the gentamicin-induced kidney injury group. Furthermore, the increased urinary albumin seen in the gentamicin-treated group might be ascribed to glomerular injury caused by gentamicin, which allows albumin leakage in the glomerular filtrate to pass to the urine when the proximal tubular reabsorptive capacity is defective or saturated [1,7,26]. In contrast, shikonin treatment in the gentamicin-induced kidney injury group significantly reduced the increased urinary albumin and calcium in a dose-dependent manner. These capabilities may be attributed to the increased NHE3 expression, which restores the competed megalin/cubilin complex activity in the proximal convoluted tubules, as well as shikonin’s nephroprotective effect against gentamicin-induced glomerular and tubular dysfunction via PMK2 inhibition, enhanced renal antioxidant defense mechanisms, and inhibition of renal apoptosis, which restores standard glomerular filtration and tubular reabsorption [13,14].

In addition, the current study’s data revealed that repeated gentamicin injections triggered full-prone kidney injury, as evidenced by elevated glomerular and tubular damage biomarkers, urine volume and flow, KSI, and renal histological changes. Traditional kidney function indicators include creatinine and BUN, which are subjected to glomerular filtration. Furthermore, Cr is employed as an index to calculate GFR. Additionally, increased KIM-1 and urine albumin levels suggest tubular damage. KIM-1, a transmembrane glycoprotein of the proximal renal tubules, is increased during acute and chronic kidney injury; therefore, its measurement can sensitively identify proximal tubular damage. Additionally, cystatin-C, a cysteine protease inhibitor found in all cells, is prone to glomerular filtration and proximal tubular reabsorption; as a result, serum and urine cystatin-C levels can be utilized to assess glomerular filtration and renal tubular damage.

Furthermore, KIM-1 and cystatin-C are more sensitive and accurate than standard biomarkers for early identification of gentamicin-induced kidney injury [1,26,27,28]. Shikonin treatment for gentamicin-injected groups reduced glomerular and tubular damage indicators, including KIM-1, serum, and urine cystatin-C levels, as well as abrogation of histopathological changes induced by gentamicin, in a dose-dependent manner, which could be attributed to improved renal glomerular and tubular functioning due to reduction in gentamicin absorption as a result of shikonin’s restoration of regular megalin/cubilin complex endocytic activity, and thus improved kidney functioning. Furthermore, recent studies found that lowering megalin endocytic activity and expression by montelukast and low-level fluoride may minimize gentamicin-induced kidney injury as well as urinary KIM-1 and cystatin-C excretion [1,29].

SIRT1 is a NAD(+)-dependent protein deacetylase expressed primarily on renal tubular cells and podocytes, where it modulates many signaling pathways and promotes cell survival. It deacetylates critical metabolic players, boosting SOD and CAT activity, and it can also decrease inflammation by inhibiting NF-κB activity via deacetylation of the p65 subunit at Lys310 [1,30,31,32]. It is believed to be a cellular cytoprotective signal that promotes the nuclear translocation/transcriptional activity of Nrf2, resulting in the activation of downstream antioxidant signals such as HO-1. In numerous kidney injury animal models, evidence has been shown that activating the SIRT1/Nrf2/HO-1 pathway can counteract oxidative stress and apoptosis; hence, the SIRT1/Nrf2/HO-1 cascade is an essential cellular defense mechanism against ROS and apoptosis [11,27]. According to the current study, with repeated gentamicin injection, the SIRT1, Nrf2, and HO-1 levels and mRNA expressions were downregulated, as previously described in gentamicin-induced renal injury animal models, resulting in enhanced oxidative, pro-inflammatory, and pro-apoptotic processes in the renal tissue [11,33]. Meanwhile, shikonin therapy elevated SIRT1, Nrf2, and HO-1 protein and mRNA expression in gentamicin-injected groups, which is consistent with previous findings [34,35,36,37]. Moreover, the impact of shikonin on Nrf2/HO-1 signaling activation may also be connected to decreased megalin/cubilin complex expression [38].

Moreover, repeated gentamicin injections in the current study raised MDA, NO, and iNOS while decreasing GSH, SOD, and TAC, indicating renal oxidative imbalance, as previously observed in various investigations [10,11,33,39]. ROS are highly reactive oxidants that cause oxidative damage to lipids, proteins, and DNA [11,33]. However, in gentamicin-induced kidney injury rats treated with shikonin, improved kidney function and histopathological architecture were significantly related to decreased MDA, NO, and iNOS, as well as increased antioxidants, GSH, SOD, and TAC, in a dose-dependent manner. Similarly, Tong et al. demonstrated that shikonin has renoprotective effects against the high glucose-induced renal proximal tubular cell, which are mediated by reducing oxidative stress via activation of the Akt signaling pathway, with MDA reduction and increased SOD and catalase levels [16]. Additionally, shikonin’s antioxidant action is thought to be related to the activation of SIRT1/Nrf2/HO-1, as previously documented [34,35,36,37].

Inflammation, in addition to oxidative stress, plays a vital role in gentamicin-induced kidney injury. TLRs play a crucial role in the innate immune response, activating numerous inflammatory cascades such as NF-κB and MAPK to modulate cytokine and chemokine production [40]. TLR-4 signaling has been connected to organ damage caused by various drugs, including gentamicin. TLR-4 activation has been detected in the kidneys of gentamicin-injected rats, and this activation has been demonstrated to engage multiple inflammatory pathways and promote inflammatory mediator production [11,40,41]. Gentamicin enhanced TLR-4 signaling in the current study; therefore, NF-κB p65, iNOS, TNF-α, IL-1β, and p38 MAPK were considerably raised in the kidneys of gentamicin-injected rats, yet IκB-α was decreased. Additionally, increased cellular ROS can activate NF-κB, resulting in the production of many inflammatory mediators [11]. The rise in NO levels detected in the kidneys of gentamicin-induced renal injury rats was related to increased iNOS expression. Increased NO can cause DNA damage and cell death once it reacts with superoxide radicals [11,42]. p38-MAPK, like NF-κB, is activated by cytokines and cellular oxidative stress, increasing inflammatory cytokine production. Furthermore, in oxidative stress, p38-MAPK can induce NF-κB activation and subsequent TNF-α production [11,43]. Shikonin treatment of gentamicin-induced renal injury groups, on the other hand, significantly decreased the expression of TLR-4, NF-κB p65, iNOS, TNF-α, IL-1β, and p38 MAPK, as well as enhanced IκB-α, confirming shikonin’s previously described anti-inflammatory activity [15,37,44,45]. Thus, by suppressing the TLR-4/NF-κB/MAPK cascade and the consequent pro-inflammatory mediators, shikonin was protected against gentamicin-induced renal injury and inflammation.

Following endocytosis, gentamicin released from lysosomes activates the mitochondrial apoptotic pathway, resulting in the release of cytochrome c in the cytosol, decreased ATP reserves, and ROS generation [5]. Cytochrome c is regarded as an apoptosis executioner since it stimulates the conversion of caspase-3 into its active form, which cleaves functional proteins that induce cell apoptosis when released in the cytosol. Additionally, the generated ROS activates caspase-3 by lowering mitochondrial membrane potential, resulting in cytochrome c translocation to the cytosol [1,27]. The anti-apoptotic protein Bcl-2 and the proapoptotic protein Bax regulate the apoptotic mitochondrial pathway [27]. Research shows that the PI3K/Akt cascade has an antiapoptotic impact and that its absence causes apoptosis [1,27,32,44,46]. When active AKT is phosphorylated by PI3K, it phosphorylates the Bcl-2-associated agonists of cell death (Bad) and reduces its proapoptotic activity. The PI3K/Akt cascade increases antiapoptotic Bcl-2 while inhibiting proapoptotic Bax and caspase-3. Therefore, the activation of the PI3K/Akt cascade guards against apoptosis by preventing the deleterious effects of Bad [47]. In line with prior research, our findings show that cytochrome c, caspase-3, Bax, and the Bax/Bcl-2 ratio were considerably elevated, whereas Bcl-2 protein level and PI3K and Akt mRNA expression were decreased, showing that gentamicin induces apoptosis [39,48,49]. Shikonin administration to the gentamicin-treated groups, on the other hand, demonstrated a significant antiapoptotic effect via the enhancement of the PI3K/Akt/Bcl-2 cascade and reduced the expression of cytochrome c, caspase-3, Bax, and the Bax/Bcl-2 ratio in a dose-dependent manner, which is consistent with previous studies [34,35].

## 4. Materials and Methods

### 4.1. Chemicals and Reagents

Shikonin, dimethyl sulfoxide (DMSO), formalin-buffered saline, gentamicin, thiopental, hematoxylin, and eosin were purchased commercially from Sigma, St. Louis, MO, USA. However, phosphate-buffered saline (PBS) was obtained from Gibco, Waltham, MA, USA, yet the lysis buffer and nuclear lysis buffer were obtained from ThermoFisher Scientific, Waltham, MA, USA.

### 4.2. Animals

Nine-week-old Wistar rats weighing 200–250 g were used in the present study. Rats were obtained from the Animal Care Unit of Prince Sattam bin Abdulaziz University, Faculty of Pharmacy, Saudi Arabia, and housed in ventilated polypropylene cages at room temperature for 12 hours (h) light/dark cycle, with ad libitum access to water and a standard laboratory diet. The rats were acclimated for seven days before the experimental procedure. All the experiments were approved by the Research Ethics Committee of experimental animal care and use at Prince Sattam bin Abdulaziz University, Al-Kharj, Saudi Arabia (Approval Reference No. SCBR-03-2022) and conform to ARRIVE guidelines and guidelines of the National Institutes of Health guide for the care and use of laboratory animals. All efforts were made to minimize the animals’ suffering, and all animal management occurred between 8:00 am and 8:00 pm.

### 4.3. Experimental Protocol

In the present study, seventy rats were used. They were randomly and blindly allocated into seven groups of ten rats per group. Group I (CON), normal rats were administered PBS intraperitoneally (i.p.) in the volume equivalent to gentamicin. Group II (SKN) was the negative control group, in which the rats were orally treated with 25 mg/kg/day of shikonin dissolved in 1% DMSO for seven days, one h after the PBS injection. Group III (GTM) was renal injury-induced rats by i.p. injection of 100 mg/kg/day gentamicin for seven days [10]. Group IV (VEH) was renal injury-induced rats, orally treated with 1% DMSO in PBS (vehicle of shikonin) in a volume equivalent to shikonin. Groups V, VI, and VII (SKL, SKM, and SKH) were renal injury-induced rats that were given 6.25, 12.5, and 25 mg/kg/day of shikonin dissolved in 1% DMSO orally for seven days, one h after the gentamicin injection [45]. All oral treatments were administered intragastrically with the feeding syringe. Immediately after the last treatment (day 7), each rat was placed in a metabolic cage for 24 h to collect urine. The collected urine was centrifuged at 5000 rpm for 10 minutes (min), and the supernatant was collected and stored at −20 °C for further assessment of urinary biochemical parameters. Twenty-four hours after the last treatment (day 8), the rats were weighed, then anesthetized with 50 mg/kg thiopental (i.p.); afterward, blood was collected from the retro-orbital sinus, centrifuged at 5000 rpm for 10 min, sera harvested, and stored at −20 °C for the assessment of the biochemical parameters of the serum. The rats were then euthanized by cervical dislocation, their abdomens dissected, and their kidneys obtained and weighed. The left kidney was preserved in 10% formalin-buffered saline for further histopathological evaluations; however, the right kidney was divided into two halves, the first half homogenized in ice-cold PBS (pH = 7.4) to get a 10% *w*/*v* homogenate, centrifuged, and the supernatant collected. However, the pellet was resuspended in nuclear lysis buffer, the suspension was centrifuged, and the supernatant was collected. The remaining half was homogenized in lysis buffer to obtain a 10% *w*/*v* homogenate. The renal tissue homogenate and supernatants were stored at −80 °C and used for evaluation of the renal tissue biochemical, nuclear, and quantitative real-time polymerase chain reaction (qRT-PCR) parameters.

### 4.4. Evaluation of Total Protein Content

Renal tissue total protein content was evaluated as nominated by the manufacturer’s instructions of a colorimetric assay kit purchased from AssayGenei, Dublin, Ireland (Cat. # BA0168), with a 0.06 mg/mL minimum detection limit. The sample absorbances were analyzed with a Stat Fax 2100 automated plate reader, Fisher Bioblock Scientific, BP., Illkirch Cedex, France. The results were expressed as mg protein/mL renal tissue homogenate.

### 4.5. Evaluation of the Renal Functions

Serum and urine creatinine (Cr) and blood urea nitrogen (BUN) levels were determined using a colorimetric assay kit purchased from Crystal Chem., Busse Rd., Elk Grove Village, IL, USA (Cat. # 80340), and Arbor Assays, Eisenhower Place, Ann Arbor, MI, USA (Cat. # K024-H1), respectively. Cr and BUN absorbances were analyzed using the Biosystems semi-automated analyzer, BTS-350, Barcelona, Spain, and expressed in mg/dL. Moreover, the serum and urine cystatin-C levels were assessed using a sandwich Enzyme-Linked Immunosorbent Assay (sELISA) kit purchased from Elabscience, Memorial Drive, Houston, TX, USA (Cat. # E-EL-R0304). However, the renal tissue kidney injury molecule-1 (KIM-1) level was evaluated using the sELISA kit purchased from MyBioSource Inc., San Deigo, CA, USA (Cat. # MBS355395). Serum and urine cystatin-C and renal tissue KIM-1 levels were expressed in ng/mL and pg/mg tissue protein, respectively, and analyzed with a Stat Fax 2100 automated plate reader. All parameters were evaluated according to the manufacturers’ instructions. Furthermore, the glomerular filtration rate (GFR) was assessed as previously described using the following formula, GFR = [urine Cr (mg/dL) × urine flow rate (mL/min)]/serum Cr (mg/dL), where the urine flow was calculated from the following formula, urine flow rate = urine volume per 24 h/1440, and the GFR was expressed in mL/min [50]. Additionally, the kidney somatic index (KSI) was calculated using the following formula, KSI = (kidney weight/rat body weight) × 100 and expressed as a percent of rat body weight [41].

### 4.6. Evaluation of Proximal Tubule Epithelial Cells Endocytosis Function

Proximal tubule epithelial cells’ endocytosis function was assessed by evaluating the renal protein levels and mRNA expressions of megalin, cubilin, ClC-5, NHE3, and urinary calcium and albumin levels. The renal protein levels of megalin, cubilin, ClC-5, and NHE3 were assessed using the manufacturer’s instructions of sELISA kits bought from LifeSpan BioSciences, Inc., Seattle, WA, USA, and MyBioSource Inc., San Deigo, CA, USA (Cat. #. LS-F39023, LS-F25369, MBS7255247 and LS-F9690, respectively). A Stat Fax 2100 automated plate reader was used to detect megalin, cubilin, ClC-5, and NHE3 levels, and expressed as pg and ng/mg tissue protein. However, the urinary calcium and albumin levels were assessed using the manufacturer’s instructions for colorimetric assay kits purchased from AssayGenei, Dublin, Ireland (Cat. # BA0032) and BioVendor, Karasek, Czech Republic (Cat. # 638-25309), respectively. A Biosystems semi-automated analyzer was used to detect the urinary calcium and albumin levels, expressed as mg/dL and μg/mL urine, respectively.

### 4.7. Evaluation of SIRT1/Nrf2/HO-1 Pathway

The SIRT1/Nrf2/HO-1 pathway in renal tissue was evaluated by measuring NAD-dependent deacetylase sirtuin-1 (SIRT1), nuclear factor-erythroid factor 2-related factor 2 (Nrf2), and heme oxygenase-1 (HO-1) protein levels and mRNA expressions. The SIRT1, Nrf2, and HO-1 protein levels were assessed using the manufacturer’s instructions of sELISA kits bought from Antibodies-Online GmbH, Aachen, Germany (Cat. #. ABIN6975596, ABIN6963254 and ABIN6963168, respectively). A Stat Fax 2100 automated plate reader was used to detect SIRT1, Nrf2, and HO-1 levels, and expressed as ng/mg tissue protein.

### 4.8. Evaluation of Oxidative Stress Markers

Renal tissue oxidative stress markers were assessed by determining malondialdehyde (MDA), reduced glutathione (GSH), nitric oxide (NO), inducible nitric oxide synthase (iNOS) levels, total antioxidant capacity (TAC), and superoxide dismutase (SOD) activity in kidney tissue. Levels of MDA, GSH, NO, iNOS, TAC, and SOD activity in renal tissue were assessed using the manufacturer’s instructions of colorimetric assay kits bought from MyBioSource Inc., San Deigo, CA, USA, Abbexa LLC, Houston, TX, USA, and Abcam, Waltham, MA, USA (Cat. #. MBS2540407, MBS2540433, MBS2540418, abx053065, ab65329, and MBS2540402). The renal tissue MDA, GSH, NO, and iNOS levels were expressed as nmol, μg, μmol, and ng/mg tissue protein, whereas TAC was expressed as μmol/mg tissue protein and SOD activity as U/mg tissue protein. The renal tissue oxidative stress markers’ absorbances were analyzed using a Stat Fax 2100 automated plate reader.

### 4.9. Evaluation of Inflammatory Signals

Renal tissue inflammatory signals were evaluated by assessing renal tissue Toll-like receptor-4 (TLR-4), nuclear factor kappa B p65 (NF-κBp65), and p38 mitogen-activated protein kinases (p38 MAPK), IL-1β, tumor necrosis factor-alpha (TNF-α), and IkappaB-alpha (IκB-α) protein levels, and the mRNA expressions of TLR-4, NF-κBp65, and p38 MAPK. The protein levels of TLR-4, NF-κBp65, p38 MAPK, IL-1β, TNF-α, and IκB-α were evaluated using the manufacturer’s instructions of sELISA kits, bought from MyBioSource and AssayGenei (Cat. #. MBS267444, MBS2505513, MBS765087, MBS825017, MBS175904, and RTFI00897, respectively). A Stat Fax 2100 automated plate reader was used to detect the TLR-4, NF-κBp65, p38 MAPK, IL-1β, TNF-α, and IκB-α levels and expressed them as ng and pg/mg tissue protein.

### 4.10. Evaluation of Apoptosis Markers

Renal tissue apoptosis was evaluated by assessing cytochrome c, caspase-3, Bax, and Bcl-2 protein levels, p-AKT, and phosphatidylinositol 3-kinase (PI3K) mRNA expression. Renal cytochrome c, caspase-3, Bax, and Bcl-2 protein levels were measured using the manufacturer’s instructions for sELISA kits purchased from CUSABIO, Wuhan, Hubei, China (Cat. #. CSB-EL006328RA, CSB-E08857r, CSB-EL002573RA, and CSB-E08854r). The renal tissue apoptosis markers’ protein levels were analyzed by an automated ELISA plate reader, Stat Fax 2100, and were expressed as ng and pg/mg tissue protein and Bax/Bcl-2 ratio.

### 4.11. Evaluation of mRNA Expressions

To assess the mRNA expressions of megalin, cubilin, ClC-5, NHE3, SIRT1, Nrf2, HO-1, TLR-4, NF-κBp65, p38 MAPK, p-AKT, and PI3K, quantitative real-time polymerase chain reaction was utilized. Total RNA was isolated from the renal tissues using a total RNA extraction kit (RNAsimple Total RNA Kit, Tiangen Biotech, Beijing, China, Cat. #. 4992858) according to the manufacturer’s instructions. Reverse transcription was performed using an iScript cDNA Synthesis Kit (Bio-Rad, Hercules, CA, USA, Cat. #. 1708891). The cDNA obtained was amplified with a real-time PCR system (qTOWER, Analytik Jena GmbH, Jena, Germany), with the thermal cycling conditions as follows: the initial denaturation and enzyme activation at 95 °C for 3 min (1 cycle), denaturation at 95 °C for 15 seconds (s), annealing at 55 °C for 30 s, and extension at 72 °C for 30 s (40 cycles) using iQ SYBR Green Supermix (Bio-Rad, Hercules, CA, USA, Cat. #. 1708880). After the cycle, the melting curve was done to ensure no non-specific products were present (1 cycle). Relative mRNA expression was normalized to β-actin and GAPDH as an internal control to standardize the difference, and the primers’ sequences are shown in Table 2.

### 4.12. Evaluation of Shikonin’s Antibacterial Activity

The agar disc diffusion technique was employed to evaluate shikonin’s antibacterial activity and its effect on gentamicin’s antibacterial activity, as described by Azouz et al. In brief, Escherichia coli was grown in Mueller-Hinton broth and plated onto Mueller-Hinton agar plates at a concentration equal to 0.5 McFarland Standard. The first well was treated with gentamicin (10 mg), the second with shikonin (10 mg), and the third with a combination of shikonin (5 mg) and gentamicin (5 mg). The plate was incubated overnight at 37 °C before being examined for bacterial growth and zone of inhibition [1].

### 4.13. Histopathological Evaluation

Renal tissues were processed as described by Suvarna et al. Briefly, left kidneys were fixed for 24 h in 10% formalin-buffered saline, embedded in paraffin, cut into 5-μm sections, and stained with hematoxylin and eosin (H&E) for further evaluation of the histopathological changes [51]. Sections were analyzed by a blinded pathologist using a light microscope (Leica Universal Microscope, Wetzlar, Germany). Histopathological changes of ten randomly selected, non-overlapping H&E sections per slide were arbitrarily scored for the presence of tubular necrosis, cast formation, leukocytic infiltration, widening of Bowman’s capsule space, congested glomerular and interstitial blood capillaries, using a 0–3 scale, where 0 = no histological change, 1 = mild change influencing <15% of renal tissue sections, 2 = moderate change influencing 15–35% of renal tissue sections, and 3 = severe change influencing >35% of renal tissue sections.

### 4.14. Statistical Analysis

The current study’s data were analyzed using IBM-SPSS Statistics Software for Windows (IBM-SPSS, version 25, Armonk, New York, NY, USA), and the results were expressed as mean ± SD. The data were compared using two-way analysis of variance (ANOVA), followed by a post hoc Bonferroni’s test, with *p*-values less than 0.05 considered significant.

## 5. Conclusions

The current study established the effectiveness of shikonin administration against gentamicin-induced kidney injury in a dose-dependent manner by inhibiting renal oxidative stress, apoptosis, and inflammation and abrogating histopathological alterations caused by repeated gentamicin injection. Furthermore, shikonin’s ability to counteract gentamicin-induced kidney injury is mediated, at least in part, by modulating renal endocytosis, SIRT1/Nrf2/HO-1, TLR-4/NF-κB/MAPK, and PI3K/Akt cascades. As a result, shikonin is regarded as a promising therapeutic agent for treating gentamicin-induced renal injury. However, further research is necessary to determine the molecular processes behind the therapeutic benefits of shikonin in renal injuries.

## Figures and Tables

**Table 1:** Shikonin mitigates gentamicin-triggered renal dysfunction.

Parameter	CON	SKN	GTM	VEH	SKL	SKM	SKH
**Serum**							
Creatinine (mg/dL)	0.60 ± 0.08 ***	0.62 ± 0.09	3.50 ± 0.47	3.47 ± 0.64	2.90 ± 0.31 **^+++^	1.75 ± 0.19 ***^+++^	0.82 ± 0.06 ***
BUN (mg/dL)	19.98 ± 1.86 ***	19.32 ± 1.95	59.64 ± 6.61	59.16 ± 10.03	51.97 ± 4.84 ^+++^	40.17 ± 3.51 ***^+++^	30.03 ± 6.47 ***
Cystatin-C (ng/mL)	16.90 ± 1.85 ***	16.89 ± 1.32	71.94 ± 9.69	71.94 ± 10.94	58.92 ± 10.93 *^+++^	40.59 ± 8.33 ***^++^	26.00 ± 5.83 ***
**Renal tissue**							
KIM-1 (pg/mg tissue protein)	5.19 ± 0.50 ***	5.13 ± 0.42	21.50 ± 4.93	21.21 ± 4.61	17.63 ± 3.02 ^+++^	14.19 ± 2.62 ***^+^	9.405 ± 1.24 ***
**Urine**							
Urine volume (ml/24 h)	8.23 ± 0.48 ***	8.27 ± 0.76	14.48 ± 3.06	14.38 ± 3.11	12.16 ± 1.22 ^+^	11.27 ± 1.75 **	9.15 ± 0.71 ***
Urine flow (μL/min)	5.71 ± 0.34 ***	5.74 ± 0.53	10.06 ± 2.12	9.98 ± 2.16	8.45 ± 0.85 ^+^	7.83 ± 1.22 **	6.36 ± 0.50 ***
Creatinine (mg/dL)	57.24 ± 5.40 ***	57.71 ± 4.58	30.36 ± 3.08	30.50 ± 4.88	36.51 ± 3.75 ^+++^	43.04 ± 5.62 ***^++^	52.57 ± 7.54 ***
Cystatin-C (ng/mL)	14.18 ± 1.05 ***	14.07 ± 1.46	78.04 ± 14.82	78.80 ± 16.51	69.81 ± 7.43 ^+++^	52.80 ± 7.79 ***^+^	39.10 ± 5.51 ***
Albumin (μg/mL)	1.96 ± 0.36 ***	1.98 ± 0.34	12.69 ± 2.07	12.57 ± 2.93	10.02 ± 1.09 **^+++^	8.49 ± 1.20 ***^++^	5.64 ± 1.12 ***
Calcium (mg/dL)	4.29 ± 0.40 ***	4.44 ± 0.42	11.41 ± 0.89	11.52 ± 1.08	9.547 ± 1.57 **^+++^	8.65 ± 0.81 ***^++^	6.86 ± 0.68 ***
**GFR (mL/min)**	0.56 ± 0.12 ***	0.55 ± 0.09	0.09 ± 0.03	0.09 ± 0.03	0.11 ± 0.01 ^+++^	0.19 ± 0.03 *^+++^	0.41 ± 0.10 ***
**KSI (%)**	0.35 ± 0.02 ***	0.35 ± 0.03	0.49 ± 0.05	0.49 ± 0.05	0.47 ± 0.05 ^+++^	0.44 ± 0.04 *^+^	0.38 ± 0.04 ***

The results were expressed as mean ± SD. CON, normal rats administered PBS i.p., SKN, the negative control group, which were orally treated with 25 mg/kg/day of shikonin dissolved in 1% DMSO for seven days, one h after the PBS injection, GTM, renal injury-induced rats by i.p. injection of 100 mg/kg/day gentamicin for seven days, VEH, renal injury-induced rats, which were orally treated with 1% DMSO in PBS (vehicle of shikonin), SKL, SKM, and SKH, renal injury-induced rats, which were orally treated with 6.25, 12.5, and 25 mg/kg/day of shikonin for seven days, respectively, one h after the gentamicin injection. * *p* < 0.05, ** *p* < 0.01, and *** *p* < 0.001 (vs. GTM group), + *p* < 0.05, ++ *p* < 0.01, and +++ *p* < 0.001 (vs. SKH group).

**Table 2:** Primers used for quantitative real-time PCR.

mRNA	Forward	Reverse
Megalin	ACTGGGCAGCAGGAAATCTT	CGGGGCATATCCACTGAGAC
Cubilin	CTGTCCAAGGCCGTTACTGT	GATGAAAACGCCAACAGGGG
ClC-5	CTTACGCCAATGGAGATCGTAGTGG	TCTTGGTTTGCCATCTGCGCTA
NHE3	GACTGGCGTGGACTGTGTGAA	TGATACGCACATGCTTGGTGAA
SIRT1	TGTTTCCTGTGGGATACCTGA	TGAAGAATGGTCTTGGGTCTT
Nrf2	CACATCCAGACAGACACCAGT	CTACAAATGGGAATGTCTCTGC
HO-1	CTATCGTGCTCGCATGAAC	CAGCTCCTCAAACAGCTCAA
TLR-4,	AGTGTATCGGTGGTCAGTGTGCT	AAACTCCAGCCACACATTCC
NF-κBp65	TGGGACGACACCTCTACACA	GGAGCTCATCTCATAGTTGTCC
p38 MAPK	ACATCGTGTGGCAGTGAAGAAG	CTTTTGGCGTGAATGATGGA
p-AKT	AGGGCAGAATCATGAGCAAGT	AGGGTCTGCATFGGATGGCA
PI3K	AGCTGGTCTTCGTTTCCTGA	GAAACTTTTTCCCACCACGA
β-actin	GCAGATGTGGATCAGCAAGC	GGTGTAAAACGCAGCTCAGTAA
GAPDH	GGCACAGTCAAGGCTGAGAATG	ATGGTGGTGAAGACGCCAGTA

## Data Availability

The data presented in this study are available on logic request from the corresponding author.
